# Optimization of DamID for use in primary cultures of mouse hepatocytes

**DOI:** 10.1016/j.ymeth.2018.11.005

**Published:** 2019-03-15

**Authors:** Leonardo Gatticchi, Jose I. de las Heras, Rita Roberti, Eric C. Schirmer

**Affiliations:** a Department of Experimental Medicine, University of Perugia, Perugia, Italy; b Wellcome Centre for Cell Biology, University of Edinburgh, Edinburgh, UK

**Keywords:** DamID, Primary hepatocytes, Genome organization, Lamina-associated domains, Nuclear lamina, Lamin B1

## Abstract

DamID, a method to identify DNA associating with a particular protein, was originally developed for use in immortalized tissue culture lines. The power of this technique has led to its adaptation for a number of additional systems. Here we report adaptations for its use in primary cells isolated from rodents with emphasis on the challenges this presents. Specifically, we present several modifications that allow the method to be performed in mouse acutely isolated primary hepatocytes while seemingly maintaining tissue genome architecture. We also describe the downstream bioinformatic analysis necessary to identify LADs and discuss some of the parameters and their effects with regards to the sensitivity of the method.

## Highlights

• DamID adaptation to primary hepatocytes may preserve tissue 3D genome architecture.
• Growth factors, vector tropism and enhancers are needed for DamID in primary cells.
• Mitochondrial contamination can yield high background signal in primary cells.
• Signal intensity comparisons can increase calling of interesting differential LADs.

## 1 Introduction

DamID is a powerful tool complementing chromatin immunoprecipitation-sequencing (ChIP-Seq) for identifying genome regions associating with particular proteins [1], [2]. A peculiar advantage of DamID over ChIP-Seq is to identify genome regions in association with the nuclear envelope. Nuclear envelope interactions with specific genes change during differentiation and this adds an additional layer of regulation that includes tight temporal regulation on top of transcription cascades to important genes for the differentiation process [3], [4]. DamID has also identified more rapid changes occurring at the nuclear envelope for example in lymphocyte activation [5]. Thus determining nuclear envelope connections to chromatin in different tissues and developmental stages is an important currently under-developed area for understanding developmental and immune disorders as well as fundamental mechanisms of genome regulation in general. However, while the nuclear envelope has extensive interactions with chromatin, these are difficult to recover by ChIP-Seq because both nuclear envelope transmembrane proteins (NETs) and lamins are highly interconnected and inherently insoluble [6], [7], [8].

The original LaminB1-DamID gets around this by fusing the insoluble nuclear Lamin B1 protein with a bacterial Dam methylase. The fusion protein localizes to the nuclear envelope while the Dam methylase recognizes GATC motifs in the DNA in its proximity and catalyzes the addition of a methyl group to the adenine to form m6A. This kind of DNA methylation is unique to prokaryotes, and permits the identification of DNA in close proximity with the nuclear envelope in higher organisms. The use of LaminB1 is advantageous for the higher eukaryotes that express them because this protein is exclusively at the nuclear envelope due to its permanent farnesylation [9] and it is a very abundant protein, present at roughly 9.5 million copies per mammalian cell nucleus [10], [11]. The DNA labelled with m6A can then be recovered and identified using microarrays or sequencing [1], [2], [12]. To ensure the specificity of the DNA labelling in DamID, in addition to expressing the Dam methylase fused to the protein of interest, the Dam methylase is also expressed alone as a soluble nuclear protein to label DNA that is accessible throughout the nucleus, and traces of both proteins are then compared. This accounts for chromatin accessibility variation and it works particularly well when the fusion is to a nuclear envelope protein because much of the peripheral DNA appears to be less accessible to the free Dam methylase. The proteins are expressed at a very low level in the procedure to reduce background and maintain specificity. This is achieved by relying upon the leakiness of the *Drosophila melanogaster* minimal heat shock protein promoter, rather than induction. Following *in situ* labeling, genomic DNA is extracted from cells and then the uniquely methylated GATC-motifs are specifically cut by the *Dpn*I restriction enzyme. Subsequent digestion with *Dpn*II, which only cuts at unmethylated GATC sequences, facilitates the selective amplification of genomic fragments that are flanked by methylated GATC motifs after ligation of adaptors to the *Dpn*I-exposed ends. Because a wide range of fragment sizes would be expected from the genome, the resulting DNA appears as a smear on agarose gels with no clearly delineated individual bands. The amplified fragments were originally identified using whole genome microarrays, but are now more commonly identified by sequencing due its simplicity and the wide range of organisms for which the procedure is now used that include mammalian cells [1], [4], [13], *Schizosaccharomyces pombe* [14]*, Caenorhabditis elegans* [15]*, Drosophila melanogaster* [16], and *Arabidopsis thaliana* [17]. Plotting the relative enrichment of genome sequences labelled by the Dam methylase fused to Lamin B1 compared to those labelled by the Dam methylase alone identifies the genomic regions associating with the nuclear envelope, termed lamin associated domains or LADs.

A major advantage of DamID over ChIP is that DamID does not rely on the availability of antibodies and avoids issues related to epitope masking [18]. Also, DamID does not require crosslinking so the interactions between DNA and the protein of interest are more likely to be direct than in ChIP. Resolution in DamID is limited to the local frequency of GATC motifs, although this is unlikely to be a significant problem at least for mouse or human cells. In *Drosophila* GATC sites within 2.5–5 Kb of a locus where the Dam methylase is bound have been reported to be efficiently methylated [19], and GATC sites are very frequent in the mouse and human genomes with a median *Dpn*I fragment size of around 260 bp, and 99.9% under 3 Kb.

Previously, we modified the DamID procedure when we used it to follow gene positioning changes during myogenesis because we found that in muscle cells there was a very high background of mitochondrial genomes carrying similar methylation, which needed to be overcome [3]. Indeed, in many different cell types and differentiation systems additional modifications are likely to be required for effective application of the DamID approach. In this paper we present an adaptation for use of DamID in freshly isolated primary liver cells. We highlight the specific challenges the protocol presents using DamID in primary cells, provide experimental workarounds and solutions, and we describe the bioinformatic analysis necessary to define LADs including the most important parameters and their effect on the detection of LADs. Although our particular approach is focused on using Lamin B1-Dam fusions, this procedure can be adapted to other soluble proteins of interest, considering their general abundance, distribution and localization.

## 2 Materials and methods

### 2.1 Isolation and culture conditions for primary mouse hepatocytes

The specific procedure described here used C57BL/6 mice, though it should be generally applicable to different mouse strains and other mammals. The animals were maintained at a constant temperature of 22 °C, 12 h light/dark cycle, and given access to food and water *ad libitum*. All the experimental procedures were carried out in accordance with European Directive (2010/63/EU) and approved by the Institutional Animal Care and Use Committee of Perugia University and the Italian Ministry of Health. Primary hepatocytes were isolated from12 to 16-week old male mice by a two-step collagenase perfusion method, as previously reported [20], with modifications as described below. Specifically, the portal vein was cannulated with a 21G needle and the liver was perfused with a Ca^2+^- and Mg^2+^-free buffer (HBSS pH 7.4, plus 0.5 mM EGTA, 25 mM HEPES, 100 U/ml penicillin, and 0.1 mg/ml streptomycin) at a rate of 5 ml/min for 5 min at 37 °C (visual reference of perfusion procedure in mice can be found at https://bit.ly/2mf71yw). Next, collagenase solution (DMEM low glucose with 0.2 g/L CaCl_2_ and 0.01 g/L MgSO_4_, supplemented with 0.3 mg/ml collagenase I (Sigma, C0130), 15 mM HEPES, 100 U/ml penicillin, and 0.1 mg/ml streptomycin), equilibrated at 37 °C, was perfused to digest the liver (about 35 ml/animal). After digestion, gall bladder was removed and the liver was placed in a Petri dish with 10 ml of warm (37 °C) collagenase solution. Hepatocytes were released from the liver capsule by tearing liver lobes with two pairs of forceps and gently shaking the tissue under a tissue culture hood to obtain a cloudy cell suspension that then was filtered through a 70 µm nylon cell strainer (Corning®, 431751). Intact hepatocytes were washed with complete cell culture medium (William’s E medium containing 5% fetal bovine serum (FBS), 2 mM l-glutamine, 0.1 µM insulin, 0.1 µM dexamethasone, 100 U/ml penicillin, 0.1 mg/ml streptomycin, and 40 µg/ml gentamicin) by three rounds of centrifugation at 50×*g* for 2 min at 4 °C. Cells were resuspended in complete culture medium and cell count was performed by trypan blue exclusion assay. Only preparations with a viability exceeding 85%, were used for the experiments. Cells were seeded at 60–70% of confluence into dishes precoated with collagen from rat tail (5 µg/cm^2^) in complete culture medium. Cells were allowed to attach for 2 h, gently washed once with PBS, then fresh medium was added for additional 2 h. Thereafter, cells were grown in complete serum-free culture medium. The primary hepatocytes were cultured at 37 °C in a humidified 5% CO_2_ incubator with daily medium renewal. Immediately after isolation, hepatocytes were tested for mycoplasma infection by a PCR based method using genomic DNA as template (20–40 ng per reaction), as previously described [21], because the mycoplasma genome has similar methylation to that of the DamID procedure and can swamp the relevant signal. Primary hepatocyte cultures are usually limited to short-term applications due to the rapid loss of their differentiation state and proliferative potential progressively during cell culture, starting from 24 h of incubation. Considering that the eradication of mycoplasma infection with appropriate antibiotics requires longer treatment exposure, mycoplasma positive preparations were discarded.

### 2.2 Production of lentivirus in HEK293FT cells

DamID relies on the efficient transfer of a transgene expressing low levels of the protein of interest fused to a bacterial Dam methylase [2]. The preferred approach for the transfer is lentiviral transduction, which allows the insertion of DNA into the nucleus of a wide range of cell types, both proliferating and non-proliferating. Lentivirus particles are typically produced by transfection of separate plasmids encoding all the necessary lentiviral components to package the target transfer DNA that is encoded in another plasmid. Human HEK293FT cells are often used because they are easy to transfect and they have been engineered to produce large T-antigen from SV40 virus, which induces the intracellular replication of plasmids bearing the SV40 origin of replication, thus greatly multiplying the copy number of each transfected plasmid and increasing lentiviral titers. We generate amphotropic lentivirus pseudotyped with the vesicular stomatitis virus envelope glycoprotein (VSV-G), which can infect both human and mouse cells. Lentivirus with different tropisms can be generated by using the appropriate plasmids encoding for other viral envelope proteins.

For each experimental condition three lentiviruses are required: 1) protein of interest, in our case LaminB1, fused to the bacterial Dam methylase, 2) Dam methylase alone, and 3) empty vector, a technical control. Comparing the signal levels from Dam-LaminB1 against soluble Dam alone controls for differences in accessibility to chromatin and sequence-dependent biases during subsequent PCR amplification. The empty vector control is very important, as in mammalian cells there should not be any other source of m6A than the DNA methylated by the exogenously introduced Dam-encoding constructs. The presence of amplified DNA in the empty vector control is often an indication of mycoplasma contamination or some other source of prokaryotic infection. If this happens, prokaryotic DNA would comprise a very high proportion of the recovered methylated DNA, severely compromising the experiment.

#### 2.2.1 Culture conditions

HEK293FT cells (Clontech) were grown at 37 °C and 5% CO_2_using high glucose DMEM supplemented with 10% fetal bovine serum, 6 mM l-Glutamine, 50 U/ml penicillin, 0.05 mg/ml streptomycin, and 0.5 mg/ml Geneticin (to maintain expression of large T-antigen). Cells were split by trypsinization 1:4–1:6 when they reached 80–90% of confluence. It is important to avoid letting the cells get too confluent and to ensure the cells are actively dividing to maximize transfection efficiency.

#### 2.2.2 Transfection

Non-replicative self-inactivating lentiviruses were generated by cotransfection of psPAX2 (lentiviral packaging plasmid), pMD2.G (VSV-G envelope protein) and transfer vectors encoding either a bacterial Dam methylase fused to LaminB1 (pLgw Dam-LaminB1), Dam methylase alone (pLgw Dam) or an empty vector (pLgw-empty) as a control. Lentiviral plasmids and the DamID backbone vectors are available at Addgene. Transfer vectors used here were a gift from Bas van Steensel. This is a 2nd generation lentiviral system is sufficiently safe for most purposes. It's worth noting, however, that there are other systems (3rd and 4th generation) that confer increased levels of safety. The drawback is that they typically yield lower viral titres because they require the co-transfection of a larger number of plasmids. The user should consider the nature of the gene of interest and consult their local regulations before deciding whether a 2nd generation system or a safer alternative should be employed.

For each transfection we prepared one 1.5 ml microfuge tube containing 4.6 µg of psPAX2, 2.8 µg of pMD2.G and 7.5 µg of transfer vector in 1mlOpti-MEM, and one 15 ml tube with 36 µl of Lipofectamine 2000 in 2 mlOpti-MEM. Following a 5 min incubation at room temperature, the DNA solution was added to the Lipofectamine, vortexed briefly, and incubated at room temperature for 15–20 min. Meanwhile, HEK293FT cells were harvested, counted with a hemocytometer, and seeded in 10 cm dishes at 6 × 10^6^ cells per dish, in 5 ml of culture medium. It is not necessary to omit antibiotics during transfection, but it may help to obtain higher yields. After incubation, the lipofection mix was added to the cells, still in suspension, mixed gently, and the dishes placed back into the incubator overnight.

#### 2.2.3 Lentivirus harvest and storage

Transfection medium was removed after 16–18 h and 12–14 ml of fresh culture medium added to each 10 cm dish. The supernatant was harvested 48 h later and centrifuged for 15 min at 500×*g* to remove cellular debris. The supernatant was carefully aspirated into fresh tubes, LentiX Concentrator (Clontech) added in a 3:1 ratio, mixed gently, and incubated overnight at 4 °C. Lentivirus particles were then precipitated by centrifugation at 1500×*g* for 45 min, and resuspended in 200–400 µl Opti-MEM. The lentiviral solutions were divided into 20–50 µl aliquots and stored at −80 °C. It is important to avoid freeze-thaw cycles to ensure the viability of the virus. Alternatively, instead of using polyethylene glycol (PEG) based reagents such as LentiX that allow the use of benchtop centrifuges most commonly found in tissue culture labs, it is possible to precipitate the lentivirus by ultracentrifugation, as described [3], [21].

### 2.3 Optimization of lentiviral transduction

To perform lentiviral transduction of primary mouse hepatocytes under optimal conditions for successful application of the DamID protocol, cells are initially tested for tolerance to polybrene, a transduction enhancer, and for the reporter gene expression levels (e.g., EGFP). Polybrene favors lentiviral particles association with target cell plasma membranes by reducing negative charge repulsion forces. However, excessive amounts can lead to apoptosis, so it is important to test for toxicity first and use a concentration that does not cause harm to cells (at least in the relatively short period of the infection), and that it does not interfere with the experiment in some other way. There are other transduction enhancers that can be used, such as protamine sulphate, retronectin, DEAE-dextran, and various proprietary commercial others. In fact, we preferred protamine sulphate in the case of mouse myogenic differentiation system because polybrene appeared to interfere with differentiation [21].

#### 2.3.1 Assessment of polybrene toxicity

Freshly isolated hepatocytes were seeded into 24-well plates (0.1 × 10^6^ cells/well) and after 4 h they were incubated with serum-free culture medium in the presence or in the absence of epidermal growth factor (EGF, 40 ng/ml) and increasing polybrene concentrations (0–10 µg/ml). The medium was replaced daily by fresh medium supplemented with EGF and polybrene. Cell morphology changes were periodically monitored by direct observation with a light microscope; images were acquired by a phase-contrast microscope equipped with a digital camera. After 72 h of incubation in the presence of polybrene, cell viability was assessed by using a modification of the 3-(4,5-dimethylthiazol-2-yl)-2,5-diphenyl tetrazolium bromide (MTT) reduction assay, which has been applied to assess cytotoxicity in hepatocytes [22], [23]. Cells were plated into 6-well plates at a density of 0.5 × 10^6^ cells per well and, after the treatments, they were detached by trypsinization. Recovered cells were resuspended in 0.5 ml of PBS containing 0.5 mg/ml of MTT and incubated for 1 h at 37 °C with gentle shaking. After centrifugation (5 min at 300×*g*), cell pellets were dissolved in 1 ml of dimethyl sulfoxide (DMSO) by pipetting and cellular debris were removed by further centrifugation2 min at 13000×*g*. Absorbance of the reduced MTT product (formazan) was measured at 595 nm by a UV–VIS spectrophotometer (UVmini-1240, Shimadzu).

#### 2.3.2 Analysis of transduction efficiency

To test the efficiency of transgene expression in isolated mouse hepatocytes, attached cells were grown for 18 h in culture medium enriched with EGF (40 ng/ml), polybrene (6 µg/ml), and EGFP-encoding lentiviral particles previously made using pRRLSIN.cPPT.PGK-GFP.WPRE (Addgene). Transduction medium was then removed, hepatocytes were washed with PBS, and wells were replenished with fresh culture medium containing EGF on a daily basis. For the analysis of exogenously expressed protein by immunoblotting, cells were seeded on 6-well plates (0.5 × 10^6^ cells/well) and cultured in 0.8 ml of transduction medium per well with the addition of different amounts of lentiviral preparation (0, 20 µl, 50 µl, 100 µl, and 160 µl). Whole cell protein lysates were obtained at 72 h after transduction by the direct addition of 150 µl per well of boiling lysis buffer (60 mMTris-HCl pH 6.8, 10% glycerol, 0.1% SDS, 0.1% triton X-100, 2 mM EDTA, and 1x protease inhibitor cocktail). After protein quantitation by Bradford assay, samples were adjusted to 2% SDS, 1.5% β-mercaptoethanol, and 0.05% bromophenol blue, subjected to SDS-PAGE (30 µg of protein per lane), and transferred to nitrocellulose or PVDF membranes. Filters were probed overnight with anti-GFP (Roche, 11814460001; 1:1000) or anti-β-tubulin (Proteintech, 66240-1-lg; 1:5000) primary antibodies and afterwards with a HRP-conjugated goat anti-mouse secondary antibody (Bio-Rad, 1706516; 1:5000). Proteins of interest were visualized using ECL reaction and images were acquired by a ChemiDoc™ imaging system. To directly visualize EGFP-derived fluorescence in transgene-expressing cells, hepatocytes were grown on 12 mm coverslips (Knittel Glass) inserted into 24-well plates (0.1 × 10^6^ cells/well) and transduced for 72 h as described above, with 1/8 of starting medium volume of the lentiviral preparation encoding for the EGFP. After the incubation period, cells were fixed with 4% PFA and quenched in 50 mM ammonium chloride for 15 min at room temperature [24]. Nuclei were stained with Hoechst 33,342 (Thermo Fisher Scientific, H3570) and coverslips mounted on ProLong™ Diamond antifade mountant (Thermo Fisher Scientific, P36970). The slides were analyzed with a confocal microscope system (Zeiss LSM 710, Oberkochen, Germany) through the excitation of EGFP at 480 nm and the acquisition of emitted fluorescence at 510 nm. The use of the EGFP reporter to visualize transduced cells is merely a quick but crude method to adjust viral titre production from batch to batch, as that is where most of the variation in the lentiviral production seems to occur, due to variations in cell culture and transfection conditions. It is crude, because different constructs will lead to different lentiviral production efficiencies. However, the DamID technique seems robust enough when driving the LaminB1-Dam expression from the uninduced inducible heat shock protein promoter, that a relatively wide range of viral titres can be used and exact titre calculations are not needed. If one batch appears to require significantly more EGFP lentiviral supernatant in order to achieve a comparable level of transduction to another batch, adjusting the amounts of the rest of the supernatants within the same batch accordingly seems to work well. In order to minimize variations, it is recommended to prepare good quality lentiviral vector DNA in large amounts and store in aliquots.

### 2.4 DamID-experiment

#### 2.4.1 Preparation of DamID cells

Cells were isolated as outlined above, seeded into 6-well plates at a density of 0.5 × 10^6^ cells per well, allowed to adhere for 4 h, and transduced overnight with serum-free complete culture medium containing 40 ng/ml EGF, the predetermined amount of polybrene (6 µg/ml) and empty, Dam alone-, orDam-LaminB1-encoding lentiviruses (transduction medium). The transduction medium was then replaced with fresh serum-free complete medium with EGF and transduced hepatocytes were left in the incubator for a further 48 h to ensure sufficient Dam-fusion protein expression and, therefore, methylation of the DNA sequences interacting with Lamin B1.

#### 2.4.2 Genomic DNA extraction

After 72 h of transduction, genomic DNA was extracted by using the DNeasy blood and tissue kit (Qiagen, 69504) according to manufacturer’s instructions. Briefly, cells were trypsinized, centrifuged at 300×*g* for 5 min and resuspended in 0.2 ml of PBS. RNAseA (0.1 mg/ml) treatment was performed for 3 min prior the addition of cell lysis buffer. Complete cellular digestion was obtained through the incubation with proteinase K (30 min at 56 °C). Digested samples were loaded onto spin columns for genomic DNA purification. Elution of genomic DNA from the columns was performed twice with 100 µl of buffer AE (preheated at 65 °C) followed by incubation (1 min at room temperature) and centrifugation at 6000×*g* for 1 min. Next, DNA was precipitated by the addition of 0.1 volumes of 3 M sodium acetate (pH 5.4), 10 µg of glycogen, and 3 volumes of 100% ethanol. Samples were incubated at −80 °C for 30 min and DNA was pelleted at 13000×*g* for 15 min at 4 °C. DNA pellets were washed with 400 µl of 70% ethanol by centrifugation at 13000×*g* for 5 min, at 4 °C. Air-dried DNA was resuspended in 13 µl of TE-lowE (10 mMTris-HCl pH 8.0, 0.1 mM EDTA) buffer at 56 °C for 1 h. DNA concentration was determined by a microplate reader (Infinite® 200 PRO, TECAN) equipped with a NanoQuant plate.

#### 2.4.3 Enrichment of Dam-methylated DNA

DNA methylated by the exogenous Dam constructs can be specifically amplified by PCR, after processing it to obtain DNA fragments flanked by methylated GATC sequences to which adaptors have been ligated. The whole process has been designed to be performed using a single PCR tube to minimize losses. For each experiment 3 aliquots of 2.5 µg are required. First, two aliquots of 2.5 µg of genomic DNA are digested overnight at 37 °C with 10 units of *Dpn*I in a total volume of 10 µl using restriction buffer 4 from NEB. *Dpn*I only cuts GATC sequences when the adenine is methylated. An additional 2.5 µg DNA aliquot should be set in a parallel reaction lacking *Dpn*I, as a control. This control should not generate any significant amount of amplification. If it does, it would suggest widespread DNA fragmentation has occurred, such as apoptosis-induced DNA cleavage and the samples should not be used. Note that a pretreatment with alkaline phosphatase could be used to prevent ligation of the DamID adaptors to the damaged DNA, and thus its amplification, however we recommend this only as a last resort if a new sample cannot be easily obtained, because a large amount of apoptotic cells in the sample could affect the resulting DamID profile. After digestion, *Dpn*I is inactivated by heating to 80 °C for 25 min, and then the dsAdR adaptor (made by annealing oligos AdRtCTAATACGACTCACTATAGGGCAGCGTGGTCGCGGCCGAGGA and AdRbTCCTCGGCCG) is ligated to the *Dpn*I digested DNA. This is achieved by adding 2 µl of 10× T4 ligase buffer (NEB), 5 units of T4 DNA ligase (NEB), dsAdR adaptor to 2 µM, and nuclease-free water to a final volume of 20 µl, and incubating at 16 °C for 16 h. Omit the T4 ligase on one of the duplicate *Dpn*I positive reactions. This control is to ensure that amplification is not due to dsAdR-independent PCR of similar sequences that might be present in the genome. If the yield of DNA is low and there is not enough for all the controls, this one is probably the safest one to omit. Ligase can be inactivated by heating at 65 °C for 20 min. Unmethylated GATC sequences are then digested using *Dpn*II (which cuts only unmethylated GATC) by adding 5 µl of 10x*Dpn*II buffer (NEB), 10 units of *Dpn*II (NEB), and nuclease-free water to make up a total of 50 µl, and incubated at 37 °C for 1 h.

At this stage, DNA fragments that were flanked by methylated DNA can be enriched by PCR, as they should have a dsAdR adaptor at both ends. Transfer 5 µl of the products (about 250 ng DNA) to a fresh PCR tube, and add 1.25 µl of 50 µM AdR-PCR primer (GGTCGCGGCCGAGGATC), 1 µl of 10 mM dNTP mix, 5 µl of 10× PCR buffer and 1 µl of Advantage PCR enzyme mix (Clontech 639105), and nuclease free water to 50 µl. We have tried other PCR reagents but this specific kit is superior to anything else we have tried. Run the following program: [10 min @ 68 °C] × 1, [3 min @ 94 °C; 5 min @ 65 °C; 15 min @ 68 °C] × 1, [1 min @ 94 °C; 1 min @ 65 °C; 10 min @ 68 °C] × 4, [1 min @ 94 °C; 1 min @ 65 °C; 2 min @ 68 °C] × 18. Run 3 µl of each reaction on a 1% agarose gel to assess quality and amplification yield. On a good experiment we expect to see a smear, typically between 200 and 1500 bp, on the products from Lamin B1-Dam and Dam alone, and nothing on any of the negative controls. If the negative controls are clear, then the amplified DNA is cleaned up using the Qiagen QIAquick PCR purification kit and the DNA quantified. How much DNA is required for sequencing will depend on the company used to carry it out and the sequencing platform they use. We typically pooled together the products from 4 to 5 PCRs as described above, and purified the DNA together. This way we obtained more than the 2.5 µg that our chosen sequencing facility required. More or fewer PCRs may be required in order to meet the requirements of different sequencing facilities. It is important to keep the number of cycles relatively low in order to avoid excessive bias towards more abundant sequences inherent to PCR. For this reason we do not recommend increasing the number of cycles stated here.

### 2.5 DNA sequencing and data analysis

We have performed LaminB1-DamID experiments with as few as 20 million 'clean' reads for human or mouse genomes with some degree of success, although we would recommend at least 30–40 million minimum. By clean reads, we mean the number of sequences obtained per sample after filtering contaminants and sequences that map to multiple regions in the genome. Noisy data due to suboptimal sequence coverage makes analysis more complicated and reduces reliability, which makes the use of multiple replicate experiments more important. One way to deal with noise is to apply local smoothing, but that reduces the spatial resolution. There is no substitute for a healthy number of good quality sequence reads to obtain good quality results.

#### 2.5.1 Sequencing data processing

The DNA obtained at step 2.4.3 was sent for sequencing offsite. We have generally used Beijing Genomics (BGI), and prepared the DNA according to their specifications. For example, using the Illumina HiSeq 2000 platform in conjunction with 90 bp read lengths, we submitted 2–2.5 µg of DNA per sample. Sequencing libraries were prepared by BGI by randomly fragmenting the DamID DNA and ligating sequencing adaptors to the ends. We have been able to obtain up to 45 million reads per sample, running four samples multiplexed per well. These details will depend on the sequencing platform and facility used and so it is important to calculate expected read depth before engaging sequencing. Some facilities will provide sequence data that have already been filtered to remove low quality reads in addition to the raw data, and some will just provide the raw sequences. Raw data can be easily custom-filtered using the Java tool FastQC (http://www.bioinformatics.babraham.ac.uk/projects/fastqc). Sequences from each sample were then mapped to the mouse genome mm9 NCBI build 37 using the Burrows-Wheeler Aligner (BWA), and sequences mapping to mitochondria or to ambiguous locations were eliminated [25]. It is typically expected to use the latest build, but note that if comparing to previously published DamID experiments it is important to also for those comparisons match by using the same build.

For analysis, we quantified 'normalized counts' per genomic *Dpn*I fragment as the proportion of the total reads in that sample that overlap with each fragment. This is done for both LaminB1-Dam and Dam-only samples. The signal for each *Dpn*I fragment is then represented as the log_2_ ratio between LaminB1-Dam and Dam-only normalized reads, followed by quantile normalization of the ratios using the R/Bioconductor package Limma [26]. The results can be output as a BED format file variant. This is a text format that contains genomic coordinates, as well as optional labels and numerical data corresponding to the signal values per fragment. While these files are simple to understand, they are also cumbersome, so to facilitate displaying data they should be converted to a compressed binary format such as BigWig. This can be achieved with a tool such as 'wigToBigWig' (https://www.encodeproject.org/software/wigtobigwig). The resulting files can be imported into the Integrated Genome Viewer (IGV, https://software.broadinstitute.org/software/igv) for visualization, where LADs appear as regions with positive signal, separated by interLADs. The data can appear ‘noisy’ both when sequence coverage is suboptimal and due to aspects of the biology of the experiment. The 'noise' is obvious when zooming into interLADs which then may appear to have frequent spurious positive signal fragments. The reason the noise is not apparent when zooming out is that DamID profiles appeared smoothed on the screen. The best way to deal with noisy data is to produce better data, but that is not always an option for financial or technical reasons. In that case, it is possible to apply gentle smoothing at the cost of losing some spatial resolution and dynamic range. Considering that the median *Dpn*I fragment size in mouse is 260 bp and that 95% are under 1238 bp, a smoothing factor of 20 would be reasonable, where the value assigned to each *Dpn*I fragment is substituted by the average of the 20 fragments surrounding it. This would lose only a few Kb of resolution but the cleaner profiles make LAD detection easier. It is important to consider that in smoothing the data as indicated, *DpnI* fragments with high read counts are treated just the same as *DpnI* fragments with low read counts, whose quantitation is less reliable. While this can work well enough, as illustrated here, there are other alternatives that one could consider. For example, smoothing before normalization or quantifying the reads using bins of a constant size rather than *Dpn*I fragments, can produce good results too and may be less influenced by the mix of high and low read count fragments. Experimenting with different sized bins is recommended, but we find that 2–5 Kb bins is usually a good starting point.

#### 2.5.2 Determination of LADs

LADs can be visually observed using a browser like IGV. LADs are thought to typically range from a few tens of kilobases to several megabases long, and cover up to 50% of the genome. This does not necessarily mean that half of the genome is located at the nuclear periphery of the nucleus at any one time, but it is probably a reflection of the variability in LADs between individual cells in the population [27]. To determine the coordinates of LADs, there are various peak finding tools available. We prefer the circular binary segmentation (CBS) algorithm used in the Bioconductor package DNAcopy [28]. DNAcopy will use the BED files generated in Section 2.5.1 and segment the genome into subregions of equivalent signal, producing another BED file as output. This output needs to be processed in order to obtain the final list of LADs: remove genomic gaps, apply threshold, merge regions and remove small regions. All these operations are performed on large but simple text files using very simple code in R with the help of tools from Bedtools, a software suite that provides a wide range of functions for the manipulation of BED files [29]. First, CBS does not take into consideration gaps in the reference sequence, so it is necessary to remove those, otherwise artifactual LADs will be generated that span large regions for which no information exists. Genomic gap tables were downloaded from the UCSC genome browser and gaps removed from the CBS output using 'bedtools subtract'. Then, a threshold is used where anything below it is eliminated. A threshold of zero is commonly used, but it can be useful to experiment with values up to 0.1 or higher, especially if the data are noisy. Next, the genomic coordinates that CBS produces may contain overlaps. Merging overlapping regions can be performed with 'bedtools merge', which also allows to specify whether to merge regions that do not overlap but are closer than a user-defined distance. We find that merging values of 5 to 8 Kb give good results without resulting in unusual fragmentation of big LADs. At this point, there are usually a large number of very small putative LADs. Some of these may be genuine, but the smaller the LAD, the lower the confidence level. Having experimental replicates becomes thus very important if the smaller LADs are to be considered. Without replicates, we usually sacrifice the smaller LADs in order to maintain confidence in our data, cutting anything below 5–30 Kb, depending on how noisy the data are. The user must investigate the effect that various parameters have on the data, and choose what appears most reasonable. If there are regions whose positions have previously been demonstrated by FISH, they can provide some useful controls in order to choose the various parameters above.

## 3 Results and discussion

**Fig. 1:**
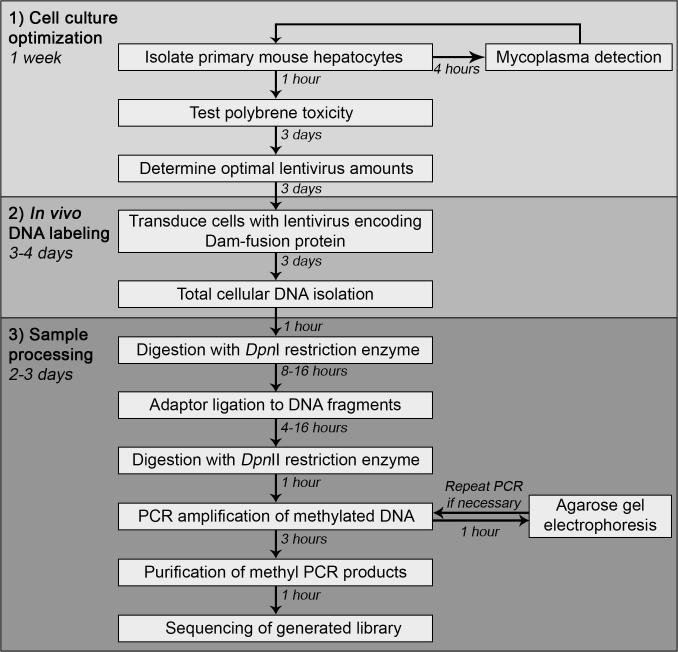
Schematic representation of DamID-Seq method applied to primary mouse hepatocytes. A cell culture optimization step (1) was included to find the optimal culture conditions to obtain the efficient methylation of target DNA sequences in cells transduced with Dam-encoding constructs (2) and straightforward sample processing (3).

The original DamID procedure was developed for use with tissue culture cancer cell lines [2]. We previously found that several modifications were needed for an *in vitro* muscle differentiation system due to high backgrounds of methylated DNA from mitochondria that accumulate within invaginations of muscle nuclei as well as several other factors [21]. In applying DamID to primary hepatocytes isolated from adult mouse livers we encountered the need for several additional modifications to the DamID procedure which we describe here. The application of the Lamin B1-DamID assay in primary cells allows the determination of the peripheral genome organization peculiar to cells in tissue as this may differ from that of cancer cell lines grown in perpetuity in 2D monolayers. Here, we present a detailed workflow from cell isolation to sequencing data analysis (Fig. 1) which enables to use tissues instead of cell lines for the application of the DamID technique in mammalian cultures, and, for the first time, specifically in primary mouse hepatocytes.

### 3.1 Primary mouse hepatocyte culture optimization

**Fig. 2:**
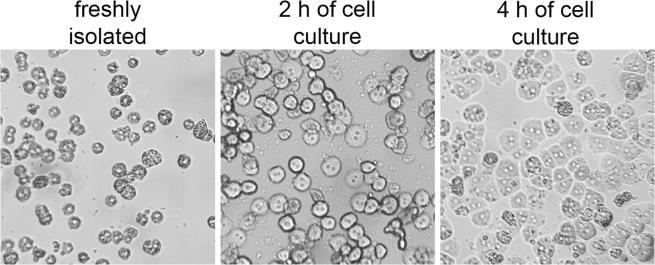
Phase contrast images of primary hepatocytes. Cells were observed right following seeding or after 2–4 h of culture. Objective magnification, ×10.

In order to successfully apply the DamID protocol in primary mouse hepatocytes, a proper isolation technique should be mastered to produce viable cultures. A detailed methodology and equipment description have been published [20], [30], [31]. The whole purification procedure, from the animal anesthesia to cell plating, was completed as quickly as possible (in 1–1.5 h) for the purpose of obtaining a sufficient number of viable cells. Freshly isolated hepatocytes were observed under a light microscope immediately after the isolation, and following 2 or 4 h of culture (Fig. 2). By this time cells had sufficiently recovered from the stress of isolation and showed luminous and round-shaped cytoplasm, clear and distinct nuclei (mostly binucleated) and the cuboidal morphology that resembles liver tissue organization. Plating the cells at 5 × 10^4^/cm^2^ allowed to reach the confluence and to form a monolayer after 24 h of culture, which is crucial to maintain hepatocyte phenotype and metabolic activity. The original DamID procedure requires maintenance of the cell cultures typically for three days after transduction for sufficient Dam-fusion protein expression and for achieving optimal Dam activity and labeling of specific DNA loci. Hepatocytes isolated for *in vitro* studies are prone to losing their defined differentiation state progressively with the time of culture, as can be determined by their obtaining a more fibroblast-like aspect by 72 h. To prevent loss of differentiation, at least for the duration of DamID experiments, the culture medium used was optimized for maintaining liver-specific functions of hepatocytes by removing serum and supplementing with insulin, dexamethasone, and EGF, which helped in slowing the dedifferentiation process and in increasing culture longevity [32], [33], [34], [35]. Particularly, EGF exposure was critical in the application of the DamID protocol in hepatocyte cultures on the basis of its strong mitogenic and anti-apoptotic activity in liver cells [36], [37], [38], [39]. The spontaneous formation of apoptotic cells in hepatocyte cultures could produce competition by apoptotic DNA fragments in the adaptor ligation and subsequent PCR amplification steps of methylated sequences. For this reason, the T4 ligase-minus control is recommended. In addition, the promotion of mitotic expansion in hepatocytes (otherwise quite quiescent when cultivated *in vitro*) permitted the correct maintenance/reassembly of chromatin architecture and localization of expressed Dam-tagged protein of interest. The absence of mycoplasma infection was detected by performing a specific PCR amplification protocol on isolated genomic DNA, which ensures enough sensitivity even for low infection levels [21]. The presence of contaminating m6A methylated DNA derived from the prokaryote can otherwise interfere with the analysis of specific methylation marks generated from the exogenously expressed Dam-fusion proteins that bind to target sequences and thus testing here revealed no mycoplasma in the isolated cells.

### 3.2 Determination of optimal transduction conditions for Dam activity in primary mouse hepatocytes

The DamID technique has the potential to be adapted to any cell line or animal into which the Dam-expressing constructs can be efficiently delivered. The differentiation status of a particular cell type strongly influences the ability of the cell to uptake exogenous DNA. In general, fully differentiated cell types are much more difficult to be genetically engineered than proliferating and undifferentiated cell types. Protocols for applying the DamID method in a variety of immortalized cell lines or primary cells have already been described, but none for primary mouse hepatocytes yet [2], [21]. Despite the presence of some reports in the literature, primary hepatocytes are relatively resistant to most plasmid DNA transfection procedures due to their lack of proliferation capacity *in vitro*, which precludes foreign DNA to access the nuclear transcriptional machinery that for most methods requires nuclear envelope breakdown in cell division. Electroporation-based methods, such as nucleofection, could be used as an alternative for hard-to-transfect cells, although with low efficiencies (<5%) and high toxicity in primary hepatocytes (personal observations). For this reason, we optimized a lentivirus-based transgene delivery for our DamID purpose, taking advantage of the charge negating agent polybrene and the high transduction efficiency, even in non-dividing cells, of VSV-G pseudotyped lentiviral particles encoding for native Dam or Dam-Lamin B1 fused proteins. Lentiviral transduction was also chosen over transfection because plasmid DNA generated in most routinely used bacterial strains will also harbor the m6A methylation so that its amplification would be likely to outcompete the specifically DamID methyl-tagged sequences from the genomic DNA during the DamID processing of samples. In fact, even Dam plasmids isolated from Dam-negative strains usually harbors detectable amounts of m6A due to leaky expression of the Dam construct. Transduction of both human and murine primary hepatocyte cultures has been already established for basic and clinical studies by using lentiviruses at high titer [40], [41], [42], [43].

**Fig. 3:**
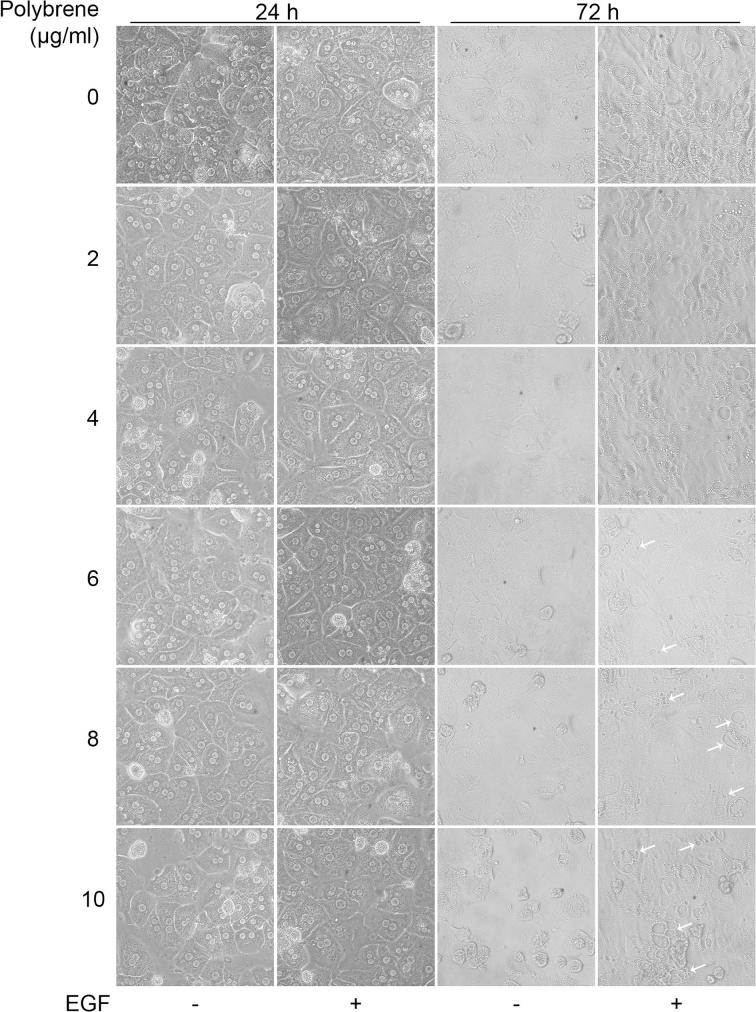
Assessment of polybrene toxicity in primary mouse hepatocytes. Cells were cultured for 72 h in the absence or in the presence of EGF with increasing amounts of polybrene (0–10 µg/ml). Cell morphology was evaluated under a phase contrast microscope and images were taken at the indicated times. Arrows indicate cytoplasmic vacuolization. Images magnification, ×20.

**Fig. 4:**
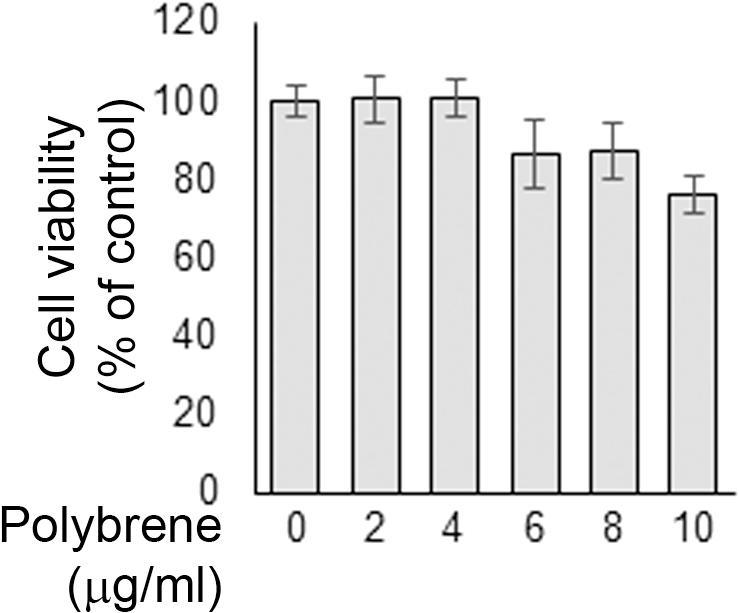
MTT reduction assay on polybrene-treated primary mouse hepatocytes. Cells were grown in the presence of EGF and treated with increasing concentrations of polybrene (0–10 µg/ml) for 72 h. Hepatocytes viability was assessed in cell suspension by the spectrophotometric measurement of MTT dye reduction (Abs at 595 nm). Results are the mean ± SD of three independent experiments in duplicate.

We investigated the effect of the transduction enhancer polybrene in primary hepatocytes by the observation of changes in cellular morphology and metabolic activity. Polybrene has been shown to markedly increase transduction efficiency but it can also negatively affect cell viability at high concentration [44], [45], [46]. After 24 h of treatment with polybrene there were no major signs of cell damage, even with the highest concentration (Fig. 3, first and second columns). When hepatocytes were subjected to longer incubation times in the presence of polybrene (up to 72 h), cell death was observed from the 8 µg/ml concentration if a standard growth medium was utilized (Fig. 3, third column). Conversely, when EGF was added to culture medium cells were protected from polybrene toxicity as shown by the preservation of hepatic functionality in the shape of intracellular accumulation of albumin granules (Fig. 3, fourth column) [31]. Indeed, the cell monolayer was maintained, although cytoplasmic vacuolization started to appear in a dose-dependent fashion from 6 µg/ml of added polybrene (Fig. 3, arrows). Accordingly, only after 72 h of treatment at 10 µg/ml polybrene, cells showed significant reduction in cell viability (>20%) as assessed by MTT reduction assay, even in the presence of EGF (Fig. 4). Hence, 6 µg/ml dose was selected for transduction.

**Fig. 5:**
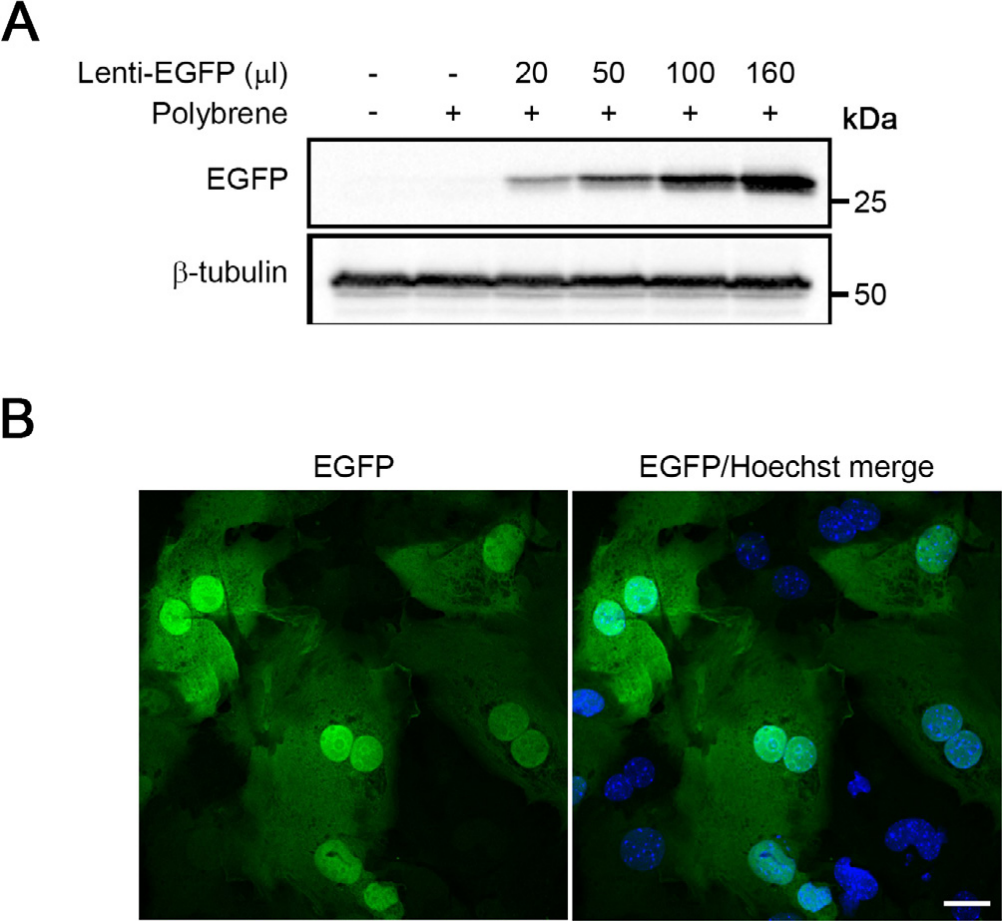
Determination of transduction efficiency of lentiviral vectors in primary mouse hepatocytes. (A) Western blot analysis of EGFP expression in cells transduced for 24 h with different amounts of lentiviral-EGFP vector (1/40, 1/16, 1/8, and 1/5 of starting medium volume), in the presence of polybrene (6 µg/ml), and cultured for additional 48 h. β-Tubulin was used as loading control. (B) Confocal fluorescence microscopy analysis of hepatocytes transduced for 72 h, as described above, with the EGFP-encoding lentiviral preparation (1/8 of starting medium volume). Images magnification, ×40. Scale bar is 20 µm.

To evaluate gene transduction efficiency in primary mouse hepatocytes, we obtained a lentiviral vector expressing the fluorescent protein EGFP, under the control of the human phosphoglycerate kinase (hPGK) promoter. Isolated cells were tested for transgene expression after 72 h of transduction with different amounts of highly concentrated lentiviral preparations by Western blot and confocal fluorescence microscopy analyses. Following transduction, hepatocytes were viable and showed that the expression levels of the EGFP reporter protein correlated with the increase of the volume of lentiviral particles added to the culture medium (Fig. 5A). Moreover, approximately 35–40% of cells were EGFP positive as observed by confocal fluorescence microscopy (Fig. 5B). In addition to the aforementioned properties, EGF addition to the culture medium markedly enhanced transgene expression following lentiviral transduction, as previously described [43], [47]. Transduction efficiency might be improved through the generation of more specific lentiviral particles, for example carrying the envelope SV-F (Sendai virus F env) protein, which provides a specific tropism for hepatic tissue [42], [44], [48]. However, evidence of successful DamID experiments in cell cultures with a transduction efficiency around 15% have been reported [2].

Another important aspect is that expression levels of the Dam fusion proteins should be very low, nearly undetectable by Western Blot or fluorescence microscopy, to avoid the production of a noisy background signal or cellular toxicity as a result of the Dam methylase activity. For this reason, weak promoters for Dam-encoding vectors should be considered like the *Drosophila* minimal heat shock protein promoter available from the van Steensel lab [49]. Results clearly demonstrated that lentiviral vector transduction itself did not impair primary hepatocytes cellular viability and it can be applied for performing DamID-Seq experiments. After these optimizations, the conditions were engaged for obtaining sufficient material from DamID cells for downstream procedures and analyses. Using four wells of a 6-well plate, 5–10 μg of genomic DNA were recovered following the purification step. The Qiagen DNAeasy purification kit is highly recommended for DamID. All the steps are performed in one tube, starting from 2.5 µg per sample, of which only a small proportion will be methylated. The yield from this kit may be lower than other methods we have tried, but the purity of the DNA obtained and the consistency of the downstream reactions make it the optimal method for the present DamID protocol.

### 3.3 Sequencing and initial data processing

**Fig. 6:**
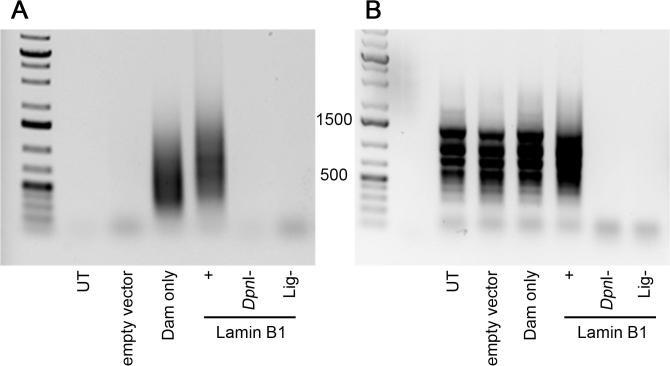
Examples of DamID PCR amplification smears. A) A smear between 200 and 1500 bp is observed only on Dam-only and LaminB1-Dam positive samples. The empty DamID vector control is negative because there is no m6A-methylated DNA available for *Dpn*I cleavage. Dam-methylated DNA that was not digested with *Dpn*I (*Dpn*I- control) also fails to amplify as there are no significant number of broken DNA ends for the DamID adaptors to ligate to, confirming the integrity of the DNA prior to treatment. Similarly, the non-ligase (Lig-) control is also negative. An additional optional control using DNA from untransduced cells (UT) is also negative. B) In contrast, when the transduced cells are infected with mycoplasma, a characteristic banding pattern is observed in all of the samples, except the Dpn- and the Lig- controls, as the mycoplasma sequences outcompete the rest. The presence of products in the UT controls shows that the contamination was present in the cells before the experiment started.

Sequencing depth is probably the single major determinant of the quality of the data. The quality and amount of DNA submitted for sequencing plays an important role in ensuring the quality of the sequences obtained. When DNA amounts are suboptimal this can often result in a large number of redundant sequences produced from a single amplicon PCR amplification. This can sometimes be observed as prominent discrete bands within the normal smear, although not always. A normal pattern of amplification is shown in Fig. 6A. Among the non-reference sequences obtained, it is common to find adaptor concatemers that have to be removed. In most cases this does not present a big problem, but when the starting amount for DamID is smaller than required this can be significant. In addition to these technical issues, there are two kinds of 'contaminants' that can make obtaining the desired sequence coverage difficult: mycoplasma and mitochondria. We would recommend that mycoplasma contamination is checked by PCR before proceeding to DNA processing for DamID as indicated earlier (Section 2.1). If there is mycoplasma DNA in the sample, it will be preferentially enriched and can easily contribute over 95% of the sequences recovered, rendering the experiment useless. For that reason, if mycoplasma is detected, we recommend discarding experimental samples and starting again. A typical DamID pattern for mycoplasma contaminated cells is shown in Fig. 6B.

**Fig. 7:**
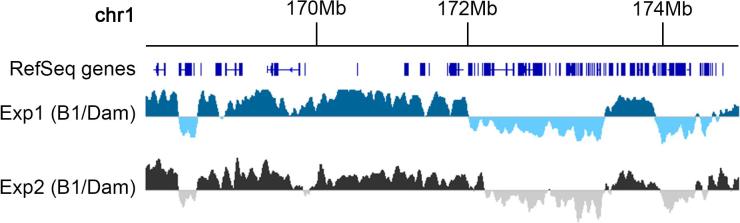
Example genome browser (IGV) representation of Lamin B1-DamID in primary mouse hepatocytes under two different conditions (Exp1 and Exp2). The genome is represented as individual *Dpn*I fragments with signal corresponding to the value log2 (Lamin B1-Dam/Dam alone). This generates a pattern of LADs (positive signal) separated by interLADs. DamID profiles shown after smoothing by 20 fragments.

**Fig. 8:**
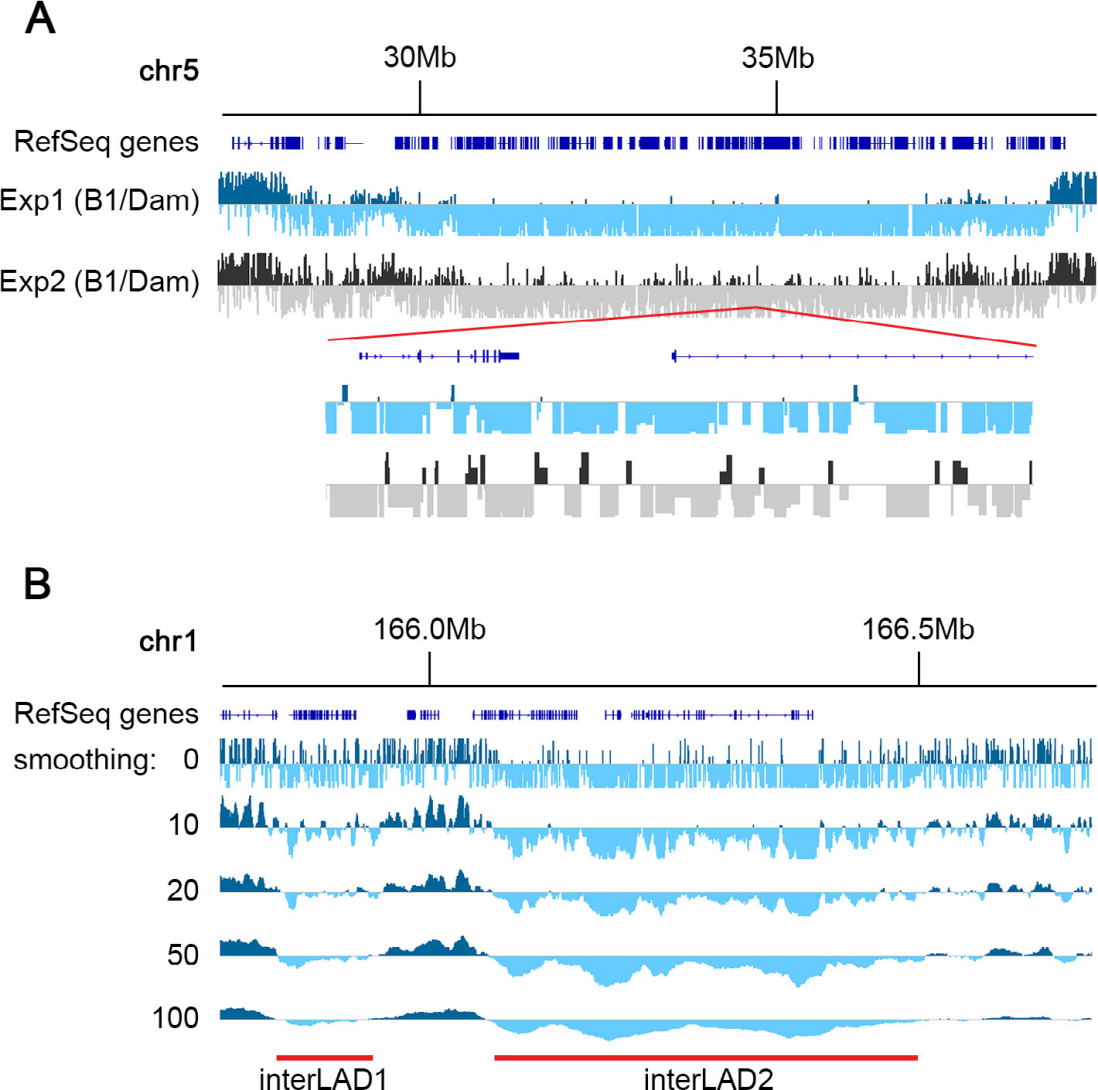
Effect of data smoothing. A) Exp2 is considerably noisier than Exp1, due to the relatively small number of clean reads, which is pushing the limits at only 20 million for Lamin B1-Dam, and 13 million for Dam alone. Large strong LADs are easily determined visually, but weaker LADs are harder to distinguish from the background. B) Effect of smoothing on DamID profiles containing a strong and a weaker interLAD. Without smoothing (smoothing = 0) the shorter and weaker interLAD1 (∼100 Kb) contains a cluster of genes and shows a clear dominance of negative signals (pale blue) yet there are a lot of interspersed positive signals that get blended into the flanking LADs. A gentle smoothing factor of 10 fragments reveals the interLAD clearly. The boundaries do not shift noticeably even at smoothing factors of 100. The longer interLAD2 (∼450 Kb) also cleans up considerably. (For interpretation of the references to colour in this figure legend, the reader is referred to the web version of this article.)

Mitochondrial DNA is also sometimes recovered together with genomic DNA. In our hands, mitochondrial sequences can comprise between 7 and 26% of the total number of sequences recovered from primary mouse hepatocytes. The reasons for this variability are not clear to us, but the amount of mitochondrial DNA co-purified with genomic DNA seems to be reproducibly higher in primary cells than in tissue culture cancer cell lines. For example, in LaminB1-DamID experiments performed with human fibrosarcomaHT1080 cells [50] mitochondrial sequences always represented less than 2% of the total, while similar experiments in the mouse myoblast cell line C2C12 produced between 20% and 74% of mitochondrial sequences [3]. It is difficult to predict how bad the mitochondrial contamination will be in any given system, so it helps to perform a pilot experiment with a small number of samples to assess this, in order to better judge what depth of sequencing is required. When sequencing depth is suboptimal, it can visually manifest as noise on the DamID profiles. This is most noticeable on long interLADs, where instead of a uniform long tract of negative values, a few isolated fragments give strong positive signals. We have analyzed a single experiment of each of two conditions using primary hepatocytes, which we simply call Exp1 and 2 (Fig. 7). Exp1 produced nearly twice the number of useful sequences as Exp2 after filtering out ambiguous mapping and mitochondrial sequences (Table 1). Although the DamID profiles are sufficiently good in both, the profile in Exp2 is noisier and more jagged than Exp1 (Fig. 8A). The signal for each experiment can be smoothed by substituting the value of each *Dpn*I fragment by the average of n fragments surrounding it. This cleans up the profiles, but it also reduces dynamic range and spatial resolution. The median *Dpn*I fragment size is 260 bp, with the 95th percentile at just over 1.2 Kb, meaning that even smoothing by 20 fragments, we should only be a few Kb off when calculating boundaries, which will typically make little differences as LADs tend to be tens to hundreds of Kb long. Fig. 8B shows the effect of increased smoothing on a noisy interLAD.

**Table 1:** Lamin B1-DamID sequencing data in primary mouse hepatocytes. Despite a healthy initial number of sequence reads, mapping quality was variable, as was the number of contaminant mitochondrial sequences. 'n total': number of sequences received from BGI; 'n chrs': number that map to reference chromosomal alignments; 'n chrM': number of mitochondrial sequences; '% chrM': percentage of mapped sequences that are mitochondrial; 'n final': number of sequences that map unambiguously to the reference genome excluding mitochondria. All numbers are in millions.

		n total	n chrs	n chrM	% chrM	n post filter
Exp. 1	Lamin B1-Dam	49.8	44.0	5.1	11.5	38.8
	Dam-alone	48.7	26.3	1.9	7.2	24.4
Exp. 2	Lamin B1-Dam	35.6	25.9	5.9	22.8	20
	Dam-alone	38.4	18.8	5.0	26.4	13.8

### 3.4 Determination of LADs

**Fig. 9:**
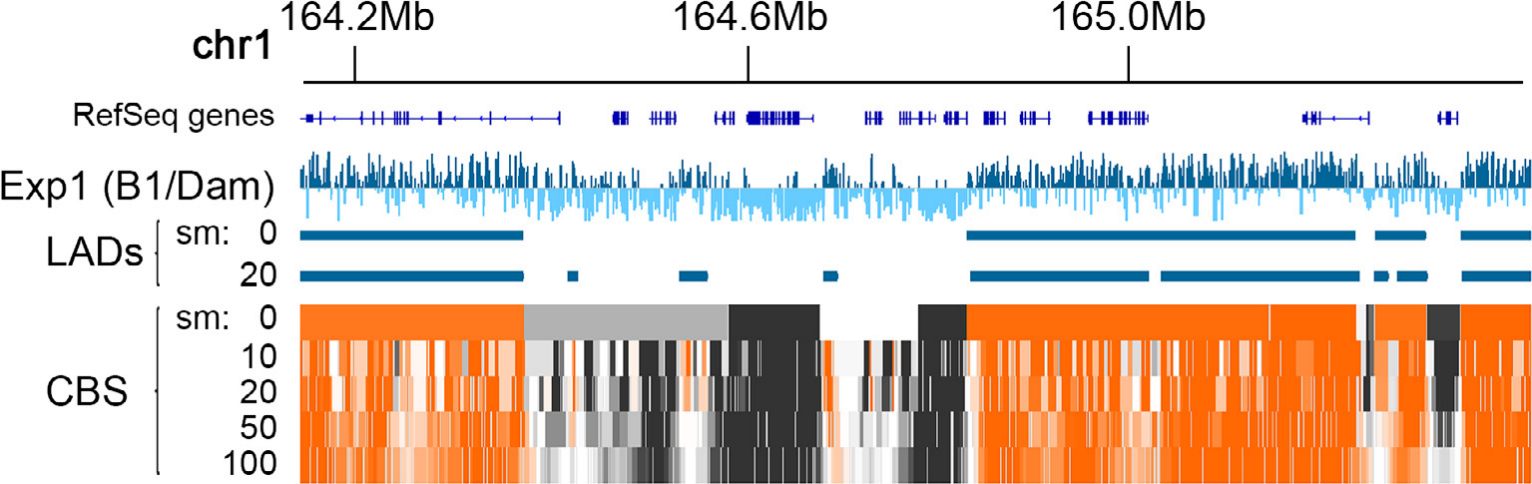
Lamin B1-DamID profile for mouse primary hepatocytes over a 1.2 Mb stretch of chromosome 1. The unsmoothed DamID profile is shown where positive values are dark blue and negative values pale blue. LADs calculated using CBS without smoothing (sm = 0) misses the smaller LADs and has in general a coarser level of resolution. Using a smoothing level of 20 (sm = 20) easily detects the smaller LADs that would otherwise be ignored. The bottom panel displays the segmentation produced by CBS using various levels of smoothing. Orange segments represent positive LaminB1 associations (LADs), and dark grey negative ones (interLADs), with white being neutral. (For interpretation of the references to colour in this figure legend, the reader is referred to the web version of this article.)

LAD coordinates are generally determined from DamID profiles (representing the ratio of Lamin B1-Dam to Dam alone signal) using a peak finding algorithm. There are many suitable for the task, each with their own pros and cons. For example, Spatial Clustering for Identification of ChIP-Enriched Regions (SICER) was created mostly to identify histone modification marks in chromatin, which are generally regions much smaller than LADs and with sharper boundaries [51]. This method has been used successfully for the detection of LADs although it produces a large number of small sized peaks that require careful post-processing, which can often result in missing some of the more subtle LADs [3], [13]. Another method, Enriched Domain Detector (EDD), was created for the purpose of detecting LADs, and excels at detecting larger genomic regions [52]. However, in our hands it is not sensitive enough to detect reliably weaker LADs, and many of these weaker LADs often change during processes that include cellular differentiation and lymphocyte activation [3], [5], [50]. In addition, it requires BAM files as input (a file format containing compressed sequence alignment information), which restricts the kind of manipulations that are easily performed on the data, including normalization options. In our opinion, the circular binary segmentation algorithm (CBS) in the Bioconductor/R package DNAcopy, originally designed to identify genomic regions with abnormal copy number by comparative genomic hybridization (CGH), gives the best results and it is also relatively straight forward to install and use [28].CBS can be used on smoothed or unsmoothed data. It works by segmenting the genome into regions of quasi-uniform signal. The size of those fragments is much larger when using unsmoothed data, which is well suited to the detection of large LADs. However, applying CBS on smoothed data can allow for better detection of smaller/weaker LADs (Fig. 9). Smoothing data slightly to better discern patterns over the underlying background noise, in addition to using CBS for detection of LADs, can be especially important when comparing LADs between two conditions. This is because sometimes strong LADs can be interrupted by small regions losing association with the nuclear periphery, and a less sensitive method could miss these.

In order to compare LADs between two or more conditions, the accepted norm has been to use a binary comparison: absence/presence of LAD, to obtain all differential regions. The success of this approach is highly dependent on an accurate determination of all LADs. An alternative approach would be to compare signal intensity differences between conditions within a window of a given length running along each chromosome. The biological meaning of the signal intensity became clearer when the DamID method was adapted to work in single cells [27]. DamID intensities can be interpreted as a proxy for the probability that a certain genomic region is located at the nuclear periphery during the experiment, in any cell of the population. High signals would indicate regions that are strongly and consistently associated with the nuclear envelope across the cell population, while weaker signals would correspond to regions that have a peripheral association in a subset of the cells. Furthermore, it has been shown in an *in vitro* differentiation system that DamID signals exhibit a reasonably strong inverse correlation with their radial distance to the nuclear envelope [4]. This means that a qualitative binary analysis (LAD vs. interLAD) is a very useful starting point, as the existing literature clearly shows, but the value of the quantitative information should not be forgotten as it can be far more sensitive to detect changes in radial positioning.

## 4 Conclusion

In this work, we describe a DamID-Seq procedure optimized for LADs identification in primary mouse hepatocytes. This approach has enabled us to profile *in vivo* spatial nuclear chromatin architecture in mouse liver cells. This methodology could be useful in order to recognize key regulators of hepatic metabolic functions and could be directly applied to assess global changes in chromatin organization and gene positioning in primary hepatocyte cultures derived from liver-specific NETs knockout mice.

## 5 Points to remember

1) The presence in the culture medium of supplements like insulin and dexamethasone during the washing steps and culture maintenance helps in slowing the dedifferentiation process typically displayed by isolated hepatocytes.
2) The addition of EGF to hepatocyte culture medium induces the cells to undergo at least one cycle of replication to allow monolayer formation and to maintain liver-specifc organization of chromatin architecture. Furthermore, EGF enhances the transduction efficiency and, accordingly, the expression of Dam-Lamin B1 fusion protein to the nuclear envelope, which results in specific DNA labeling by Dam on target DNA sequences.
3) Lentiviral preparations could be used at higher titer since they showed low cellular cytotoxicity in primary hepatocytes.
4) Due to the high autofluorescence of hepatocytes we suggest to quench PFA fixed samples as described in materials and methods section and more importantly we encourage the indispensable use of a confocal microscope to acquire fluorescence images by finely selecting excitation and emission wavelenghts for EGFP channel.
5) It is highly recommended to use up to four wells for each condition when performing genomic DNA extraction for DamID experiments, to ensure the collection of enough material for downstream processing.
6) It is important to use the DNeasy blood and tissue kit. In our experience the DamID results are extremely variable when using phenol–chloroform extractions to isolate DNA.
